# Retraction Note: Aged bone matrix-derived extracellular vesicles as a messenger for calcification paradox

**DOI:** 10.1038/s41467-026-77218-4

**Published:** 2026-08-27

**Authors:** Zhen-Xing Wang, Zhong-Wei Luo, Fu-Xing-Zi Li, Jia Cao, Shan-Shan Rao, Yi-Wei Liu, Yi-Yi Wang, Guo-Qiang Zhu, Jiang-Shan Gong, Jing-Tao Zou, Qiang Wang, Yi-Juan Tan, Yan Zhang, Yin Hu, You-You Li, Hao Yin, Xiao-Kai Wang, Ze-Hui He, Lu Ren, Zheng-Zhao Liu, Xiong-Ke Hu, Ling-Qing Yuan, Ran Xu, Chun-Yuan Chen, Hui Xie

**Affiliations:** 1 Department of Orthopedics, Xiangya Hospital, Central South University, Changsha, China; 2 Movement System Injury and Repair Research Center, Xiangya Hospital, Central South University, Changsha, China; 3 The Second Xiangya Hospital, Central South University, Changsha, China; 4 Xiangya Nursing School, Central South University, Changsha, China; 5 Department of Laboratory Medicine, Affiliated Zhejiang Hospital, Zhejiang University School of Medicine, Hangzhou, China; 6 Department of Emergency Medicine, Xiangya Hospital, Central South University, Changsha, China; 7 Department of Sports Medicine, Xiangya Hospital, Central South University, Changsha, China; 8 National Clinical Research Center for Geriatric Disorders, Xiangya Hospital, Central South University, Changsha, China; 9 Hunan Key Laboratory of Organ Injury, Aging and Regenerative Medicine, Changsha, China; 10 Hunan Key Laboratory of Bone Joint Degeneration and Injury, Changsha, China

Retraction Note: *Nature Communications* https://doi.org/10.1038/s41467-022-29191-x, published online 18 March 2022

The Authors have retracted this Article following the identification of multiple irregularities in the figures and data, including but not limited to,

- partial duplication of the field of view between Figs. 3j and 7f
- duplicated quantitative values in several histology-related quantitative panels (Fig. S4d/Fig. S8e and Fig. 7i/Fig. 7j)
- duplicated serum ion concentration values in the calcium and phosphorus datasets (Figs. S6a, S6b, and S16b)

These issues affect multiple figures and both image-based and quantitative data in the Article. While the authors maintain confidence in the overall conclusions of the study, these errors compromise the integrity of the published data. To uphold the rigor of the scientific record, the Authors have therefore decided to retract this article.

Hui Xie has stated on behalf of all authors that they agree with the retraction.

